# People Living With Human Immunodeficiency Virus During the COVID-19 Pandemic: Experiences With Telemedicine

**DOI:** 10.1177/15248399211001071

**Published:** 2021-03-12

**Authors:** Dagan Coppock, Christine Quimby, Jonathan Nunez, Cynthia Whitener, John Zurlo

**Affiliations:** 1 Thomas Jefferson University, Philadelphia, PA, USA; 2 Penn State College of Medicine, Hershey, PA, USA

**Keywords:** coronavirus, COVID-19, HIV, mobile health primary care, telemedicine

## Abstract

Preserving routine primary care for people living with human immunodeficiency virus (PLWH) has been an important challenge during the COVID-19 pandemic. Telemedicine platforms have offered novel means through which care for these individuals may be maintained. Opt-In for Life is a unique mobile health application that contains telemedicine capabilities as well as other features designed specifically for the care of PLWH. Opt-In for Life was implemented early in the pandemic at Hershey Medical Center, although the center is now using a different telemedicine platform across its health care system. Institutional decisions regarding telemedicine platforms are complex. Opt-In for Life contains features that may improve the care of PLWH where telemedicine software alone may be limited.

## Assessment of Need

Since the emergence of the COVID-19 pandemic, maintaining the standards of primary care in the United States has been a challenge. This has been of particular concern with regard to the routine care of PLWH (Jiang et al., 2020). HIV clinics have experienced reductions in office hours, limitations to the quantity of provider visits, and restrictions on face-to-face appointments, all of which have led to a disruption in the continuum of care (Qiao et al., 2021). One way of addressing these barriers is the use of mobile health technologies, including those with telemedicine capabilities.

## Description of Innovation

OPT-In for Life is a mobile technology–based intervention designed to improve HIV care for people living with human immunodeficiency virus (PLWH; Zurlo et al., 2020). In addition to a number of features that aid in education and medication adherence, the OPT-In for Life application (app) also has a Virtual Visit feature, capable of supporting telemedicine appointments. This feature allows human immunodeficiency virus (HIV) care team members to conduct a secure video conference with patients at a convenient time or place, thus providing uninterrupted HIV primary care. Furthermore, it allows patients to request a virtual visit with different care team members, including providers, registered nurses, or case managers. Though the app was rolled out prior to the onset of the COVID-19 pandemic, its inclusion of telemedicine capabilities was prescient. In this Practice Note, we summarize our experience using the Virtual Visit feature, including successes and challenges, both before and during the COVID-19 pandemic.

## Challenges and Successes

The HIV program at Penn State Health Hershey Medical Center launched the Opt-In for Life app’s Virtual Visit feature in early 2020. The feature served as a videoconferencing platform in which providers and PLWH could engage in a virtual office visit in a secure, Health Insurance Portability and Accountability Act–compliant setting. At the time of the Virtual Visit feature’s pre-COVID rollout, our clinicians had little experience with video-enhanced patient communication. Because of this as well as the lead time necessary for providers to learn the technology, uptake of the Virtual Visit feature was initially low. However, interest in the technology was facilitated through demonstrations of the feature to the HIV program staff. Additionally, during routine clinic hours members of the Opt-In for Life administrative support team made themselves available to walk each staff member through the entire Virtual Visit process in real time.

As use of the Virtual Visit feature expanded, patients provided positive feedback regarding appointment ease and access to care. Barriers such as transportation costs, patient work hours, and household obligations were reduced by the convenience of the Virtual Visit feature. Beyond the telehealth capability, providers and patients were able to utilize the app’s other features, including secure instant messaging and HIV-specific educational content, both of which were shown to contribute to patient retention and viral load suppression (Zurlo et al., 2020). Having these features and telemedicine bundled into one app allowed patients to manage multiple aspects of their care using one source.

Despite these early successes, the Opt-In for Life app faced an institutional impasse. Although the app and its Virtual Visit feature existed prior to the pandemic and was adopted early on, Hershey Medical Center contracted with a telemedicine company for the use of a platform separate from Opt-In for Life. This platform allowed for ease of billing and scheduling across the institution, but it lacked the features of Opt-In for Life that had been shown to improve HIV outcomes. The Hershey Medical Center ultimately required clinicians to use the organization’s app, rather than Virtual Visit feature, for telehealth.

## Implications for Replication and Next Steps

Since the onset of the COVID-19 pandemic, telemedicine platform options have increased significantly (Wosik et al., 2020). Consequently, health care centers have had to choose platforms carefully based on operational costs, billing requirements, data privacy, and equitable access (Ortega et al., 2020). These issues are a particular challenge for large institutions such as Hershey Medical Center. Creating a uniform, health care system–wide telemedicine platform is a reasonable approach to address the potential pitfalls associated with this evolving approach to healthcare. However, PLWH in our region may benefit from other features offered by the Opt-In for Life app.

With regard to unmet needs in our region, smaller HIV provider practices may not have the resources to create their own platform. Opt-In for Life may be able to serve that need. For example, the Northwest Alliance serves metropolitan Erie as well as a large surrounding rural population of PLWH. The Northwest Alliance struggled during the first wave of the pandemic and is interested in exploring how Opt-In for Life may serve its population.

The potential benefits of Opt-In for Life are not limited to smaller health care centers. Thomas Jefferson University is interested in incorporating the app into its HIV program. In addition to the features that have been associated with improved outcomes, the app now has Spanish language educational content and personal reflections, which can serve the Spanish-speaking PLWH at Thomas Jefferson University.

Future research will examine how telehealth platforms, such as Opt-In for Life, may best cater to different settings. Patient engagement in telehealth platforms and how that engagement may in turn reflect HIV-specific clinical outcomes, such as antiretroviral adherence and viral load suppression, will be the aim of future research endeavors.
